# Socioeconomic differences in the benefits of structured physical activity compared with health education on the prevention of major mobility disability in older adults: the LIFE study

**DOI:** 10.1136/jech-2016-207321

**Published:** 2016-04-08

**Authors:** David Bann, Haiying Chen, Chris Bonell, Nancy W Glynn, Roger A Fielding, Todd Manini, Abby C King, Marco Pahor, Shannon L Mihalko, Thomas M Gill, the Life Study investigators

**Affiliations:** 1 Centre for Longitudinal Studies, UCL Institute of Education, London, UK; 2 Division of Public Health Sciences, Department of Biostatistical Sciences, Wake Forest School of Medicine, Winston-Salem, North Carolina, USA; 3 Department of Social and Environmental Health, London School of Hygiene and Tropical, Medicine, UK; 4 Center for Aging and Population Health, Department of Epidemiology, University of Pittsburgh, Pittsburgh, PA, USA; 5 Nutrition, Exercise Physiology, and Sarcopenia Laboratory, Jean Mayer USDA Human Nutrition Research Center on Aging at Tufts University, Boston, Massachusetts, USA; 6 Department of Aging & Geriatric Research, University of Florida, Gainesville, Florida, USA; 7 Stanford University School of Medicine, Stanford, California, USA; 8 Department of Aging & Geriatric Research, University of Florida, Gainesville, Florida, USA; 9 Department of Health and Exercise Science, Wake Forest University, Winston-Salem, North Carolina, USA; 10 Yale School of Medicine, New Haven, Connecticut, USA

**Keywords:** AGEING, PHYSICAL ACTIVITY, DISABILITY, SOCIAL INEQUALITIES

## Abstract

**Background:**

Evidence is lacking on whether health-benefiting community-based interventions differ in their effectiveness according to socioeconomic characteristics. We evaluated whether the benefit of a structured physical activity intervention on reducing mobility disability in older adults differs by education or income.

**Methods:**

The Lifestyle Interventions and Independence for Elders (LIFE) study was a multicentre, randomised trial that compared a structured physical activity programme with a health education programme on the incidence of mobility disability among at-risk community-living older adults (aged 70–89 years; average follow-up of 2.6 years). Education (≤ high school (0–12 years), college (13–17 years) or postgraduate) and annual household income were self-reported (<$24 999, $25 000 to $49 999 and ≥$50 000). The risk of disability (objectively defined as loss of ability to walk 400 m) was compared between the 2 treatment groups using Cox regression, separately by socioeconomic group. Socioeconomic group×intervention interaction terms were tested.

**Results:**

The effect of reducing the incidence of mobility disability was larger for those with postgraduate education (0.72, 0.51 to 1.03; N=411) compared with lower education (high school or less (0.93, 0.70 to 1.24; N=536). However, the education group×intervention interaction term was not statistically significant (p=0.54). Findings were in the same direction yet less pronounced when household income was used as the socioeconomic indicator.

**Conclusions:**

In the largest and longest running trial of physical activity amongst at-risk older adults, intervention effect sizes were largest among those with higher education or income, yet tests of statistical interactions were non-significant, likely due to inadequate power.

**Trial registration number:**

NCT01072500.

## Introduction

There is a recognised need to evaluate whether the benefits of community-based interventions differ by socioeconomic group.^1–3^ To the authors' knowledge, no prior study has evaluated whether the effectiveness of interventions to prevent mobility disability among older adults differs according to socioeconomic group. This is important to investigate given the substantial individual and societal costs of mobility disability,^4^ and the observed differences in disability risk across socioeconomic groups: older adults with lower education or lower income, on average, have worse physical performance levels^5^ ^6^ and a higher risk of mobility disability;^7^ ^8^ interventions may either widen or narrow these differences.^1^ ^2^

Recent findings from the largest and longest duration randomised trial of physical activity in older adults demonstrated that a structured physical activity intervention—relative to a health education intervention—reduced the incidence of major mobility disability by 18% among at-risk community-living older adults (HR 0.82, 95% CI 0.69 to 0.98).^9^ We investigated whether the benefits of this intervention differed by socioeconomic group and hypothesised that the benefits would be more pronounced among more socioeconomically advantaged groups, since greater socioeconomic resources may foster exercise participation and protect against adverse events and illnesses that could impede exercise.^10^ ^11^

## Methods

### Study sample

The Lifestyle Interventions and Independence for Elders (LIFE) study was a phase 3 randomised controlled trial conducted at eight field centres in eight US states. The rationale, design and methods of the LIFE study have been presented in detail elsewhere,^12^ as have baseline characteristics.^13^ Participants aged 70–89 years were eligible if they were at increased risk of mobility disability (ie, a Short Physical Performance Battery score ≤9), yet were able to walk 400 m at their usual pace in 15 min without sitting, leaning or assistance from a walker/another person. Participants were additionally required to be relatively sedentary (ie, reported <20 min/week in the past month getting regular physical activity and ≤125 min/week of moderate activity). The two self-reported socioeconomic indicators were education attainment (≤ high school (0–12 years), college (13–17 years) or postgraduate) and annual household income (<$24 999, $25 000 to $49 999 and ≥$50 000). In total, 1635 participants (∼67% women) were randomised, ranging from 200 to 216 per site. Ethical approval was provided by the Institutional Review Boards, and written informed consent obtained from all study participants.

The physical activity intervention consisted of moderate-intensity- walking (with a 150 min goal per week), and strength, flexibility and balance training, in supervised clinical centres and at participants' homes. The comparison arm was a health education programme comprising workshops (weekly for the first 26 weeks, then monthly thereafter) on topics (excluding physical activity) such as nutrition, healthcare systems and safe travel.

The primary outcome of the LIFE study was major mobility disability, objectively defined as loss of ability to walk 400 m. Persistent mobility disability was defined as two consecutive assessments with major mobility disability or major mobility disability followed by death. Participants were followed up every 6 months for an average of 2.6 years—from the time of enrolment and start of the interventions (February 2010 to December 2011) to the end of the interventions (December 2013).

We compared baseline demographic and health characteristics across socioeconomic groups, then compared the risk of mobility disability in each treatment group according to education or income using Cox regression models. We formally tested these differences through education or income×intervention interaction terms.^14^ ^15^

## Results

Participants with higher compared with lower education were more frequently male, and had a lower prevalence of hypertension, yet they did not consistently differ with respect to measured physical performance (table 1—income results shown in online supplementary table). The effect estimates—showing the effect of physical activity on reducing the incidence of mobility disability—were greatest among participants with postgraduate education (HR 0.72, 95% CI 0.51 to 1.03) and weaker for those with intermediate (0.81, 0.61 to 1.06) or lowest education (0.93, 0.70 to 1.24; table 2), yet CIs overlapped. These differences were similar for persistent mobility disability. The education group×intervention interaction term was not statistically significant for either outcome (p=0.54 and 0.62, respectively). Differences in effect sizes by household income were similar (table 2), and income×intervention interaction terms were also not statistically significant (p=0.90 and 0.79, respectively).

**Table 1:** Descriptive statistics of the LIFE study sample at baseline, by education group

	Physical activity group				Health education group				
Characteristic	≤ High school (K–12)	College (13–17)	Postgraduate	p Value*	≤ High school (K–12)	College (13–17)	Postgraduate	p Value*	
N (%)	273 (33.4)	345 (42.2)	199 (24.4)		263 (32.4)	338 (41.6)	212 (26.1)		
Age (years)	78.5 (5.1)	78.7 (5.4)	78.9 (5.2)	0.73	79.0 (5.1)	79.2 (5.2)	78.9 (5.4)	0.84	
Women (%)	207 (75.8)	223 (64.6)	116 (58.3)	0.001	199 (75.7)	225 (66.6)	126 (59.4)	0.001	
Race (% non-white)	85 (31.4)	68 (19.8)	37 (18.6)	0.001	65 (24.9)	47 (13.9)	40 (19.0)	0.003	
Income ≥$50k (%)	44 (18.4)	104 (33.7)	94 (53.4)	0.001	35 (15.1)	95 (32.2)	100 (51.8)	0.001	
BMI (kg/m^2^)	30.5 (5.8)	29.9 (5.7)	29.7 (5.7)	0.25	31.0 (6.2)	29.9 (6.1)	30.2 (6.5)	0.08	
Conditions (%)									
Hypertension	210 (77.2)	226 (66.1)	137 (68.8)	0.009	207 (78.7)	231 (69.0)	139 (67.5)	0.009	
Diabetes	88 (32.4)	86 (25.0)	42 (21.1)	0.02	80 (30.4)	92 (27.3)	60 (28.7)	0.80	
Myocardial infarction	25 (9.2)	24 (7.0)	11 (5.5)	0.31	20 (7.6)	27 (8.0)	21 (10.0)	0.60	
Stroke	23 (8.5)	21 (6.1)	13 (6.5)	0.51	15 (5.7)	24 (7.1)	13 (6.2)	0.77	
Cancer	55 (20.2)	73 (21.3)	50 (25.1)	0.42	49 (18.6)	76 (22.5)	65 (31.0)	0.006	
Chronic pulmonary disease	54 (19.8)	45 (13.1)	31 (15.6)	0.08	48 (18.3)	44 (13.1)	30 (14.3)	0.21	
3MSE score, 0–100 scale	89.5 (5.7)	92.0 (5.2)	93.5 (4.8)	0.001	89.6 (5.8)	91.9 (4.8)	93.7 (4.7)	0.001	
Walking/weight activities (min/week)	77.3 (134.5)	63.1 (106.8)	92.1 (140.6)	0.032	84.6 (131.3)	85.8 (137.2)	90.1 (134.4)	0.90	
Sedentary time (min/day)	628.1 (120.4)	641.2 (109.5)	678.5 (114.6)	0.001	632.7 (110.6)	645.9 (114.5)	646.0 (107.2)	0.37	
Lower light-intensity activity (min/day)	173.7 (54.5)	160.7 (51.1)	152.1 (53.0)	0.001	172.5 (51.5)	158.6 (57.1)	160.5 (52.8)	0.02	
Higher light-intensity activity (min/day)	27.7 (21.1)	27.4 (21.7)	27.0 (24.8)	0.96	28.5 (28.9)	27.6 (25.6)	28.3 (23.4)	0.93	
SPPB score	7.4 (1.5)	7.5 (1.6)	7.5 (1.6)	0.83	7.3 (1.6)	7.3 (1.6)	7.4 (1.6)	0.65	
400 m walking speed, m/s	0.8 (0.2)	0.8 (0.2)	0.8 (0.2)	0.06	0.8 (0.2)	0.8 (0.2)	0.8 (0.2)	0.50	

Results are presented as mean (SD) or as n (%).

*Comparison of education groups using ANOVA or χ^2^ tests; all accelerometry measures were adjusted for wear time; accelerometer cut points were as follows: sedentary: 100 counts/min; lower light: 100–760 light; higher light: >760 counts/min.

3MSE, Modified Mini-Mental State Examination; ANOVA, analysis of variance; BMI, body mass index; LIFE, Lifestyle Interventions and Independence for Elders; SPPB, Short Physical Performance Battery.

**Table 2:** HRs of major and persistent mobility disability for physical activity versus health education, by education or income group

	Physical activity		Health education		HR (95% CI)	p (Interaction)*
	N	Number of events (%)	N	Number of events (%)		
*Major mobility disability*						
Education						
≤High school (0–12)	273	95 (34.8)	263	93 (35.4)	0.93 (0.70 to 1.24)	0.54
College (13–16)	345	96 (27.8)	338	119 (35.2)	0.81 (0.61 to 1.06)	
Postgraduate (17+)	199	55 (27.6)	212	75 (35.4)	0.72 (0.51 to 1.03)	
Income						
≤$24 999	230	77 (33.5)	232	87 (37.5)	0.86 (0.63 to 1.17)	0.90
$25 000–$49 999	252	75 (29.8)	258	94 (36.4)	0.77 (0.57 to 1.05)	
≥$50 000	242	63 (26)	230	72 (31.3)	0.82 (0.59 to 1.16)	
*Persistent mobility disability*						
Education						
≤High school (0–12)	273	51 (18.7)	263	57 (21.7)	0.83 (0.57 to 1.21)	0.62
College (13–16)	345	41 (11.9)	338	59 (17.5)	0.68 (0.45 to 1.01)	
Postgraduate (17+)	199	28 (14.1)	212	46 (21.7)	0.63 (0.39 to 1.01)	
Income						
≤$24 999	230	36 (15.7)	232	48 (20.7)	0.78 (0.5 to 1.2)	0.79
$25 000–$49 999	252	37 (14.7)	258	50 (19.4)	0.71 (0.46 to 1.09)	
≥$50 000	242	29 (12)	230	43 (18.7)	0.62 (0.39 to 0.99)	

HR and interaction terms calculated using Cox regression.

*p Values were derived from intervention×socioeconomic group interaction terms.

10.1136/jech-2016-207321.supp1 Supplementary table

## Discussion

In the largest and longest running trial of physical activity among at-risk older adults, we found no statistically significant evidence that the benefits of physical activity on reducing the risk of mobility disability differed by education or income group. The lack of differential benefit indicates that this form of intervention, if established more widely across the country, would neither widen nor narrow socioeconomic inequalities in disability incidence. However, because intervention effect sizes were largest among those with higher education or income and because the trial was unlikely to have been adequately powered to detect statistically significant interactions, it is possible that the intervention could widen socioeconomic differences in disability incidence.

Despite the large size of the LIFE study (with 246 participants experiencing major mobility disability), it may still have been underpowered to detect genuine differences in intervention benefit across socioeconomic groups, since tests for interaction require particularly high statistical power^14^ ^15^—more power than is required to test for a main intervention effect across all participants. For example, assuming a HR of 0.9 for low education and 0.75 for high education (50% of participants in each group), and an α of 0.05 (two-sided), we calculate that 3776 events would be required to achieve 80% power to detect a statistically significant interaction (p<0.05). In order to be sufficiently powered, future studies may therefore require larger sample sizes and/or be pooled into meta-analyses. In addition, the primary outcome of this study was mobility disability—a dichotomous outcome; studies using continuous outcomes are likely to have greater power to detect differences in intervention benefit.

Socioeconomic differences in enrolment and follow-up could also lead to socioeconomic differences in intervention benefit. Minimal loss to follow-up occurred in the LIFE study (4% per year^9^), and recruitment was relatively successful (eg, 59.4% of those contacted by mail^13^). However, we were unable to investigate socioeconomic differences in enrolment due to the lack of relevant data for non-responders. Other physical activity trials have reported lower enrolment among those of lower education or income, a pattern which could widen inequalities,^16^ ^17^ yet efforts were made in the LIFE study to enable participation regardless of socioeconomic circumstances (eg, free transport provision for some participants) which may have limited this tendency.

Strengths of this study include the use of data from a large number of older adults with extensive follow-up across the USA and objective outcome assessment. Although both education and (banded) household income were available, future studies may include more refined socioeconomic measures which better distinguish socioeconomic groups and thereby enable greater power.^1^ For example, continuously measured disposable income and wealth may be especially relevant socioeconomic indicators in old age,^18^ ^19^ while early life socioeconomic conditions may have independent effects on adult physical activity and disability risk.^6^ ^20^

In the largest and longest running trial to date, we found that the benefits of a physical activity intervention in preventing mobility disability did not significantly differ by socioeconomic group, yet effect sizes of intervention benefit were largest among those with higher education or income. Our findings add to a limited number of existing studies, some of which found that physical activity interventions inadvertently increase inequalities in other outcomes at younger ages.^2^ These studies could ultimately be pooled to enable greater statistical power for subgroup interactions, and used to identify the characteristics of interventions which both improve average population health and reduce inequalities.

**What is already known on this subject**

- A large-scale multicentre randomised trial recently found that a physical activity intervention, compared with a health education programme, reduced disability incidence in at-risk community-dwelling older adults (the Lifestyle Interventions and Independence for Elders (LIFE) study).
- There is an important lack of evidence on whether health-benefiting interventions differ in their effectiveness by socioeconomic group.

**What this study adds**

- There was no statistically significant evidence that the benefits of physical activity on reducing the risk of mobility disability differed by education or income group, yet effect sizes of intervention benefit were largest among those with higher education or income.
- Despite being the largest physical activity trial yet conducted among older adults, there may have been inadequate power to detect genuine differences in benefit by socioeconomic group. Future studies may be designed to specifically evaluate potential socioeconomic differences in benefit, and/or pool estimates from multiple studies.
