# Supplementary material for: Socioeconomic differences in the benefits of structured physical activity compared with health education on the prevention of major mobility disability in older adults: the LIFE study
Source: J Epidemiol Community Health. 2016 Apr 8;70(9):930–3. doi: 10.1136/jech-2016-207321 (PMC5013156; doi:10.1136/jech-2016-207321)
Supplement: Supplementary table [file jech-2016-207321supp_table.pdf]

Supplementary Table 1. Descriptive statistics of the Lifestyle Interventions and Independence for Elders (LIFE) Study sample at baseline, by income group

	Physical activity group				Health Education group			
Characteristic	≤$24999	$25000-$49999	≥$50000	P	≤$24999	$25000-$49999	≥$50000	P
N (%)	230 (31.8)	252 (34.8)	242 (33.4)		232 (32.2)	258 (35.8)	230 (31.9)	
Age (years)	78.5 (5.1)	78.5 (5.3)	78.9 (5.3)	0.72	79.1 (5.3)	79.3 (5.2)	78.7 (5.2)	0.49
Women (%)	188 (81.7)	179 (71.0)	107 (44.2)	<0.001	181 (78.0)	172 (66.7)	119 (51.7)	<0.001
Race (% non-white)	77 (33.5)	52 (20.9)	39 (16.1)	<0.001	60 (26.1)	39 (15.2)	35 (15.2)	0.002
BMI (kg/m^2)	30.6 (5.5)	30.1 (6.0)	29.6 (5.5)	0.19	30.4 (5.9)	30.5 (6.1)	30.0 (6.3)	0.69
Conditions (%)								
Hypertension	170 (74.2)	168 (66.9)	169 (70.4)	0.22	174 (75.7)	178 (69.0)	161 (71.9)	0.26
Diabetes	64 (27.9)	72 (28.7)	57 (23.6)	0.38	66 (28.6)	79 (30.7)	69 (30.1)	0.87
Myocardial infarction	21 (9.1)	20 (8.0)	12 (5.0)	0.2	18 (7.8)	18 (7.0)	22 (9.6)	0.56
Stroke	17 (7.4)	16 (6.4)	17 (7.1)	0.9	19 (8.3)	9 (3.5)	20 (8.7)	0.04
Cancer	43 (18.8)	54 (21.5)	62 (25.7)	0.19	47 (20.3)	61 (23.6)	65 (28.4)	0.13
Chronic pulmonary disease	40 (17.4)	39 (15.6)	36 (14.9)	0.75	34 (14.8)	43 (16.7)	32 (14.0)	0.69
3MSE score, 0-100 scale	89.9 (5.8)	92.2 (5.3)	92.6 (5.0)	<0.001	90.0 (5.7)	92.1 (4.9)	93.2 (4.7)	<0.001
Walking/weight activities (min/week)	75.4 (128.5)	67.1 (124.4)	79.5 (122.8)	0.53	87.5 (138.1)	90.9 (138.5)	89.2 (133.2)	0.96
Sedentary time (min/day)	628.5 (110.7)	652.6 (120.7)	654.9 (115.3)	0.05	629.9 (106.8)	650.5 (115.6)	643.3 (102.3)	0.18
Lower-light intensity activity (min/day)	168.6 (52.2)	168.0 (53.5)	152.9 (53.2)	0.004	161.3 (48.4)	168.8 (57.6)	156.7 (54.5)	0.08
Higher-light intensity activity (min/day)	26.0 (18.4)	28.4 (24.1)	28.0 (24.4)	0.54	25.1 (21.5)	28.9 (30.2)	31.3 (27.1)	0.09
SPPB score	7.5 (1.5)	7.5 (1.6)	7.4 (1.7)	0.85	7.3 (1.6)	7.3 (1.6)	7.4 (1.7)	0.45
400m walking speed, m/s	0.8 (0.2)	0.8 (0.2)	0.9 (0.2)	<0.001	0.8 (0.2)	0.8 (0.2)	0.8 (0.2)	0.003

Note:
Results are presented as mean (SD) or as n (%);
*Comparison of education groups using ANOVA or chi-squared tests; all accelerometry measures were adjusted for wear time; accelerometer cut points were as follows: sedentary: <100 counts/min; lower-light: 100-760 light; higher-light: >760 counts/min.
Abbreviations: 3MSE, Modified Mini-Mental State Examination; BMI, body mass index; SPPB, Short Physical Performance Battery.
